# Cultivating Lifestyle Transformations in Obstructive Sleep Apnea

**DOI:** 10.7759/cureus.12927

**Published:** 2021-01-26

**Authors:** Roohi Afshan Kaleelullah, Preethi P Nagarajan

**Affiliations:** 1 Dentistry, California Institute of Behavioral Neurosciences & Psychology, Fairfield, USA; 2 Dentistry, Ragas Dental College, Chennai, IND

**Keywords:** obstructive sleep apnea, lifestyle, disorder, smoking, modifications, osa, diet, changes, therapy, exercise

## Abstract

Today, our well-being and awareness have become markedly determined by our way of living through our everyday activities. Needless to say, daily practices specifically have a significant impact on the quality of sleep. Obstructive sleep apnea (OSA) is an exhausting sleep disorder regulating an individual's routine life. Although several therapeutic modalities are available for curing OSA, behavioral therapies are also utilized for a positive outcome. Besides, several studies are performed to prove the efficacy of lifestyle strategies to resolute OSA in adults. Reducing weight, quitting alcohol and smoking, eating a nutritional diet, and exercising are the modifications to benefit people. This review aims to expand our knowledge of the association between alterations to comportment and better treatment outcomes for sleep apnea.

## Introduction and background

Obstructive sleep apnea (OSA) is a real disease identified by reduced oxygen saturation due to recurrent episodes of insufficient or complete airflow cessation in the upper airway during sleep. OSA is a condition that is unnoticed and frequently undiagnosed, even in symptomatic patients, and is predominant in 2-9% of adults. OSA is up to four times more prevalent among men and seven times more common among people who suffer from obesity with a body mass index [BMI] >30 [1].

The apnea is an abnormal breathing event terminated by arousals, which disrupts sleep perpetuation and leads to wake-time sleepiness [2]. As per the Bernoulli effect, airflow velocity peaks at the site of stricture in the airway. As the airway velocity surges, pressure on the lateral wall decreases, and the airway collapses as the transmural pressure is attained. Transmural pressure is the variation between intraluminal pressure and the pressure in the surrounding tissues. This effect also explains why there is a widespread OSA prevalence in obese patients with excessive fat deposition in necks [3].

The severity of OSA is typically assessed with the apnea-hypopnea Index (AHI), which is the proportion of apneas and hypopneas occurring every hour of sleep. Specific disease severity measures that signify the extent of average oxyhemoglobin desaturation and extent of sleep discontinuities (i.e., arousal frequency) are also used in the clinical and research fields [4]. Common symptoms of OSA include increased daytime sleepiness, blare snoring with periods of stillness followed by gasps, dry mouth, morning headaches, waking up repeatedly to urinate at night [5], impaired concentration and memory, stress, and depression [6].

## Review

Diagnosis and management 

The clinical analysis should include a comprehensive sleep history and a physical examination that incorporates the cardiovascular, respiratory, and neurologic systems [7]. Succeeding the evaluation, polysomnography, a diagnostic standard for OSA, is completed in a sleep laboratory [8]. It can demonstrate frequent apneas occurring in sleep that signal the inhibition of the sleeping brain to regulate the airway patency. Pieces of evidence showcase an abnormal pharyngeal airway that leads to inspiratory collapse as a normal loss of pharyngeal dilator muscle tone that happens with sleep [2].

Hypopneas and apnea should prevail for at least 10 seconds, wherein hypopneas are evaluated by oxygen desaturation of 3% or more or awakenings from sleep. An AHI classifies OSA based on the severity. A value of 15 or more events per hour, or five or more events per hour with existent symptoms or concomitant cardiovascular disorders, are an essential diagnostic criterion for OSA [8,9]. OSA management typically involves behavioral modifications, continuous positive airway pressure (CPAP) therapy, mouth appliances, mandibular advancement devices, supplement therapies, and surgeries in severe cases of OSA [10].

Conventional treatment 

Positive Airway Pressure Devices

PAP devices are used to reduce the AHI values and are considered primary devices to manage OSA. These devices are commonly used for treating moderate-to-severe OSA. PAP machines include a mask that is fitted over the nose and mouth or sometimes beneath the nose. It provides pressurized air that flows regularly or is suspended during sleep. An increase in air pressure avoids airway collapse and enables continuous breathing during sleep without fragmentation [11]. The types of PAP devices include CPAP, auto-titrating PAP (APAP), bilevel positive airway pressure [11,12]. Of all, the CPAP is the most common. CPAP machines need the airway pressure to be constantly between inhalation and exhalation. Such pressure is generally accomplished by a servo-regulated air compressor regulating the airway pressure as close to the desired pressure despite the patient's inspiration and exhalation [12]. As formulated by the American Academy of Sleep Medicine, the subsequent guidelines for PAP therapy have been suggested to treat patients with sleep-disordered breathing (SDB). Clinicians are recommended to use PAP instead of no therapy to treat OSA in adults with excessive sleepiness. PAP therapy is instigated either by working with only APAP at home or in-laboratory PAP adjustment in adults with OSA and no huge comorbidities. Clinicians are suggested to use either CPAP or APAP for the ongoing treatment of OSA in adults; educational interventions are required to be given with PAP therapy initiation in adults with OSA [13].

Mandibular Advancement Appliances

Mandibular advancement device (MAD) has proven to be a significant device in snoring and OSA treatment. It has been considered an aid for mild-to-moderate sleep apnea and decreasing bruxism (nocturnal grinding) [11,14]. MAD appliances, also known as the mandibular repositioning appliances, protrude the mandible forward, thereby changing the tongue position and mandible. In this way, the MAD prevents the upper airway collapse. It has been determined that the design features affect the efficiency of MAD for treating OSA [15]. MAD is considered an alternative, which can be the initial choice of treatment in simple snorers, mild OSA patients, mild-moderate OSA with low BMI, and patients suffering from the increased resistance of the upper airway syndrome and secondly preferred in patients who are intolerant to PAP devices, patients at elevated surgical risk, and who react adversely to surgical interventions [16].

Surgeries

Surgical treatment plans are devised according to the anatomical locations of the obstructions. Common surgeries include nasal surgery, redundant tissue surgery of the oropharynx, surgeries of the tongue, craniofacial surgery, tracheostomy, and bariatric surgery for weight loss. Surgical treatments are done when the benefits outweigh the risks when anatomical abnormalities are detected, and conventional treatment modalities like CPAP fail to alter the patient's quality of life with OSA [17].

Behavioral interventions

Weight Loss

Obesity has been extensively linked to OSA's occurrence, and the treatment plan includes a reduction of weight. Obesity contributes to the airway's anatomical narrowing, and research has found that a 10% weight gain can be equal to a six-fold rise in OSA risk [5,18]. Tuomilehto et al. conducted a randomized control study inclusive of a very low-calorie diet and active lifestyle counseling. Participants in the intervention group received 24 months of intervention with an intensive weight reduction program for three months on a very low-calorie diet. In contrast, the control group received three general dietary and exercise counseling sessions. The lifestyle interventions were constructive in reducing body weight (-10.7 ± 6.5 kg; BMI, -3.5 ± 2.1 [mean ± SD]), and the average change in AHI between the study groups was noticeable. The outcome was beneficial with a marked weight reduction for most patients with mild OSA and was maintained at one-year follow-up [19]. In place of the positive results, Tumilehto et al. subsequently followed a long-term observational post-interventional randomized control study aimed at the effects of weight reduction and increased physical activity on OSA's progress. There were remarkably lower AHI scores; the OSA degree declined from mild to moderate in six participants in the mediation group and 12 participants in the control group. Furthermore, the disease worsened to severe OSA in two people in the control group. Thus, the intervention achieved a 61% reduction in OSA progression than the control group [20]. 

Effects of weight management: To analyze the impact of weight management for OSA, a panel of sleep and pulmonary specialists, weight-loss experts, and behavioral scientists developed a questionnaire, reviewed the relevant resource, and approached the Grading of Recommendations, Assessment, Development and Evaluation (GRADE) method to provide a synopsis of the literature. Based on this research, evidence-based recommendations were made to manage overweight/obesity in adults with OSA. The panel made specific recommendations. The committee proposed participation in a comprehensive lifestyle intervention program consisting of a reduced-calorie diet, exercise/increased physical activity, and behavioral counseling for obese OSA patients. For the OSA patients with a BMI ≥27 kg/m^2^, who could not lose sufficient weight despite participating in a comprehensive lifestyle weight management program and have no contraindications or active cardiovascular disease, an evaluation for potential anti-obesity pharmacotherapy was suggested. For the OSA patients with a BMI ≥35 kg/m^2^, whose weight loss remained undeterred despite participating in a comprehensive lifestyle intervention program, and who had no contraindications, referral for bariatric surgical evaluation was suggested [21].

Benefits of weight loss: When weight loss is achieved, there is a homogeneous improvement in OSA severity. Furthermore, weight loss benefits may include the actual resolution of OSA, betterment or prevention of type-2 diabetes mellitus, lowering of blood pressure, and enhanced quality of life [22]. It has been suggested that behavioral approaches to weight loss have no risk; however, pharmacological and surgical therapies have related risks [23]. Castro et al. devised a randomized controlled trial (RCT) that involved individuals with moderate-to-severe OSA with AHI ≥15 events/hour. This transient, comprehensive, and community-based program consisting of physical and oropharyngeal exercises did not produce appreciable transformations in the AHI in patients with moderate-to-severe OSA who were recommended CPAP therapy. However, following the program, the walking distance capacity increased, and body weight decreased. Also, significantly low AHI scores were achieved for patients under 60 years old who followed the comprehensive intervention program [24]. Investigations were made to correlate the association between the efficacy of weight loss and improvement in OSA. According to the study, weight loss reduced the upper airway soft tissues' volumes in people with obesity and OSA. The AHI scores improved with weight loss by depletions in the tongue fat. Hence, the researchers consider new treatments that reduce tongue fat for patients with OSA [25].

Diet and Exercise

People who make the optimal dietary choices that include a neutral diet rich in fruits, vegetables, whole-grain products, and high-quality proteins and avoid refined grains, refined sugars, and highly processed foods may find it easier to control their body weight without tracking calories or constricting portion sizes daily. Exercise and energy loss play a crucial role in weight loss. A sedentary lifestyle requires reduced energy intake even when consuming a healthy diet to maintain weight loss. Specifically, the ideal strategy to achieve long-term weight loss is drifting to healthy lifestyle habits with a diet plan compatible with individual food options and lifestyle habits. Successful diets involve gradual and stable changes. An even more optimal goal is weight loss maintenance and weight loss prevention through holistic education, motivation, and behavior changes to obtain long-lasting weight loss and other health benefits [26].

Impact of various types of diets: Dobrosielski et al. investigated the effect of diet, exercise, and weight loss in obese, older adults with OSA. Participants followed a calorie-restricted diet and exercise (three days a week). The severity of OSA decreased after the exercise and weight loss program among older adults, advocating that this lifestyle approach may be an essential first-line nonsurgical and nonpharmacological treatment for OSA [27]. Of all the diets, the Mediterranean diet seems a promising approach to improve OSA severity, compared to a low-fat, calorie-restricted diet. A typical Mediterranean diet consists of the ample use of olive oil as the primary culinary source of fat, liberal intake of plant-based foods, intake of fresh and varied fruits as the typical dessert, regular intake of fish and other seafood, moderate wine consumption with meals, limited meat consumption, and intermediate consumption of dairy products [28]. This diet is also rich in antioxidants, micronutrients, and anti-inflammatory properties, possibly exerting improved upper airway neuromuscular control and reducing inflammation and oxidative stress, leading to a decrease in OSA severity. High-protein diets may promote less indulgence in overeating and inhibit muscle mass loss; formula diets and intermittent diets can be used to achieve a substantial and rapid loss of weight, but the results last for a short term. The long-term safety and sufficiency data are lacking for intermittent diets, and the optimal pattern and severity of energy restriction remain controversial [26].

Meta-analysis for diet/exercise intervention: Edwards et al. postulated a study to assess the effectiveness of diet, exercise, and combined intervention of the two for OSA treatment. The study provided a systematic review and meta-analysis of RCTs focused on the impact of lifestyle interventions for treating OSA. RCTs were determined through a systematic database search between 1980 and May 2018, and meta-analysis was followed by meta-regression. The influence of diet or physical activity as a separate entity or a combination of both on the primary (AHI) and secondary (BMI) outcomes of interest was examined through two meta-analyses. Meta‐analysis was based on whether researchers reported mean post‐intervention or mean within‐group change (pre vs. post) and mean differences of the AHI for both intervention and control groups. At the end of the first meta-analysis, a remarkable depletion in AHI was detected in all three types of interventions. After the second analysis, a noteworthy reduction in BMI was discerned in both the diet‐only and combination subgroup analyses, but not for the exercise‐only analysis. The consequence of the intervention length and baseline AHI and BMI on the modification in BMI and AHI was investigated in all studies. No significant correlation was observed in any investigated potential cofactors and changes in outcomes (i.e., changes in AHI and BMI). A meta‐regression was utilized to find whether changes in BMI results across studies explained changes in AHI results. However, it did not identify a critical association. Edwards et al. concluded that lifestyle interventions, regardless of type, effectively reduced the severity of OSA despite the fluctuating impact they have on BMI. Even though interventions often improve OSA severity, interventions alone must not be employed to resolve OSA; however, they may have ideal improvements in several patient‐related symptoms and cardiovascular, metabolic risk factors [29].

Aerobic exercise and myofunctional therapy for OSA: Exercise programs for OSA benefit patients by reducing the severity of the condition and daytime sleepiness and increasing sleep efficiency and maximum oxygen consumption. In addition to systemic clinical benefits provided by physical exercise, OSA patients engaged in a regular, predominantly aerobic exercise program have shown a reduction in disease severity and daytime sleepiness and increased sleep quality and peak oxygen consumption, irrespective of weight loss [30]. Several studies are being focused on the association between physical exercise and OSA. The patients with OSA have suppressed hemodynamic reactions to physical exercise related to the severity of the disease. One study found that routine and principally aerobic exercise training significantly reduces OSA severity, even without a significant bodyweight reduction [31]. Myofunctional therapy is a non-invasive therapy to reinforce the oropharyngeal muscles and the tongue, thereby regulating the resting tongue position. The treatment includes a list of mouth and face exercises to strengthen the musculature. Exercises should be repeated for 45 minutes each day, and the entire set should be done at least four times each day for optimal outcomes. For optimal results, exercises should be performed consistently for at least two years [32]. Several studies are performed to assess orofacial myofunctional therapy (OMT)'s efficacy as a treatment modality for OSA. Following the review of 11 studies, it was emphasized that OMT reduces snoring and severity of OSA and improves life quality. Also, patients show increased adherence to CPAP therapy when OMT is used as an adjunct therapy [33].

Smoking cessation

Many researchers have suggested potential mechanisms for the relationship between smoking and OSA. Widely presented mechanisms to describe how smoking may root OSA comprise sleep architecture modifications: nicotine-stimulated relaxation of the upper airway muscles, upper airway inflammation due to smoke inhalation, and nicotine-induced increase in sleep arousal threshold. The higher number of nicotine binding sites observed in smokers is a result of chronic hypoxia. This surge in the number of nicotine binding sites further sustains the vicious cycle of smoking and expands smoking frequency. Also, depression and mood disorders are common in patients with OSA and may represent a reason for individuals with OSA to be addicted to smoking [34].

Kyung Soo Kim et al. demonstrated that smoking caused oropharyngeal narrowing and worsened OSA severity through a study that involved 57 OSA participants. Participants were classified based on the OSA severity and smoking history; duration of smoking and histological differences were noted in the uvular mucosa. Finally, it was concluded that smoking duration and severity of OSA were interconnected. Smokers had significant changes in uvular mucosa histology caused by increased calcitonin gene-related peptide, a neuroinflammatory marker for peripheral nerves [35].

Synergistic effects of OSA and smoking: Researches have emphasized the synergistic effects of smoking and OSA as both smoking and OSA cause dysfunctions, including oxidative stress, endothelial dysfunction, and inflammatory system activation, leading to cardiovascular diseases through common pathophysiological mechanisms [36]. Nicotine chewing gum right before sleep reduces obstructive and mixed sleep apnea but does not influence central type apnea. This was because the levels of nicotine gradually decreased during the night, and the number of sleep apneas raised as a consequence of the "rebound effect." This effect has been regarded as a possible mechanism for the influence of smoking on OSA [37]. Varol et al. conducted a study that included 964 patients with a polysomnographic diagnosis of OSA. The degree of OSA severity was assessed among active and former smokers, and desaturation time during sleep was considered. AHI values were higher with lower non-rapid eye movement-3 (NREM-3) and higher NREM1-2 stages (p = 0. 017, p = 0.007, p < 0.001) in severe smokers compared to mild smokers. It was found that cigarette smoking was associated with young-age disease onset, and heavy smokers had severe OSA; male patients had severe OSA than female patients [38]. A large cross-sectional study investigated whether tobacco smoking synergized with sleep apneas and intensified metabolic profile abnormalities. Also, the study assessed whether smoking cessation improved metabolic disorder in patients with untreated OSAs. In the study, smokers were defined as self-reported smokers who smoked cigarettes for at least one year. Ex-smokers were former cigarette smokers who gave up after smoking for at least one year and remained smoke-free for at least one year. Furthermore, this study explored the combined effects of sleep apneas and smoking on metabolic parameters. Interactions between smoking and OSAs aggravated metabolic disorders after multiple adjustments. Henceforth, the authors recommend clinicians to encourage their patients to quit smoking to improve sleep apnea and prevent metabolic disorders and cardiovascular morbidities [39]. 

The relevance of smoking frequency: According to the recent study investigated by Esen et al., the effects of cigarette smoking on OSA remain uncertain. The study involved comparing non-smokers and smokers with regard to the severity of OSA. The data collected for the analysis included demographics, symptoms, medical history, AHI (REM and NREM AHI), minimum oxygen saturation, and smoking frequency. The result was that OSA severity was not correlated with smoking frequency, and severe OSA was found in male smokers and smokers with BMI <30. Hence, the researchers preach future studies with a larger sample for expanded evaluation of the association of smoking with OSA [40].

Quitting alcohol

According to the Centers for Disease Control and Prevention, heavy drinking is consuming 15 drinks or more per week for men, and heavy drinking is consuming eight glasses or more per week for women [41]. Heavy drinkers appear to be at expanded risk for OSA, distinctly when they snore, though even moderate amounts of alcohol significantly increase the frequency and severity of apneas among persons with OSA, especially in the first few hours of sleep when blood alcohol levels are the highest. Alcohol ingestion increases the duration and frequency of apnea episodes in patients with OSA and improves hypoxemia in the first hour of sleep. Alcohol consumption depresses the genioglossus muscles' respiratory activity and reduces arousal response time, thereby exacerbating OSA during sleep [42].

Effects of alcohol on sleep: Irrespective of the dosages, alcohol reduces sleep onset latency with an integrated first-half sleep and elevated sleep fragmentation in the second half of the sleep cycle. There are dose-related effects on REM sleep in the first half of sleep, with low and moderate doses showing no profound impact on REM sleep in the first half of the night. On the other hand, at high doses, REM sleep reduction in the first part of sleep is considerable. Total night REM sleep percentage is lower in most studies at moderate and high amounts, with no clear trend apparent at low doses [43].

Jung Choi et al. explored the association between habitual alcohol consumption (HAC) and metabolic syndrome in patients with SDB. The study indicated that heavy drinkers had increased BMI and waist circumference compared to the non-drinkers and light-drinkers (5.1 drinks/week). The researchers observed that the distribution of SDB severity was significantly varied concerning the HAC. Severe SDB was the highest (40.9%) in heavy drinkers than in non-drinkers or light drinkers; on the other hand, the percentage of mild SDB (AHI 5-15/hour) was the highest (42.4%) in non-drinkers. The outcome suggested that increased alcohol intake is analogous to the severity of SDB. Also, it was indicated that sleep quality declined based on HAC amount. 

Heavy drinkers had the most severe SDB (the highest AHI [32.2/hour] and the lowest oxygen desaturation [81.1%]) and the most fragmented sleep structures (the highest arousal indices [34.5/hour] and N1 sleep proportion [27.3%]) in comparison to the non-drinkers or light drinkers. It was concluded that despite fair amounts, people with HAC and SDB might have a two-fold more significant risk of developing severe respiratory-related disturbances during sleep [44]. Simou et al. followed a systematic review and meta-analysis of the link between alcohol consumption and OSA in adults. The measures of OSA severity included respiratory distress index and AHI. Analyses were on levels of alcohol consumption: light/moderate/heavy drinking; alcohol dependence; or drinks/grams of ethanol per day, week, or 24 months. Data extraction included study design, the definition of alcohol intake and the result (OSA), locality, reference group, setting, the total number of people enrolled, demographics, finding, and recognized limitations. The study demonstrated that people who consume alcohol, either about no exposure or those with relatively high intakes compared to those with low inputs, are approximately 25% more likely to have OSA. The author states that alcohol consumption worsens OSA, specifically in cases before bedtime [45].

Positional therapy

Positional therapy (PT) is a second-line treatment for patients when other treatments fail to provide help with OSA [46]. In patients who are intolerant to CPAP, PT could be a substitute for decreasing the severity of OSA, especially for patients with "supine centered OSA," assuring efficacy of PT [47]. DeVries et al. investigated the effectiveness of PT compliance through a study involving 53 patients, of which 40 patients underwent a follow-up polysomnographic evaluation under the review after a median time interval of three months. It was determined that short-term results showed that PT with the well- known tennis ball technique (both self-made frameworks, commercial waistbands) effectively decreases the time spent in supine sleeping position in patients with positional OSA. Besides, AHI, the severity of the respiratory events, and daytime sleepiness were profoundly reduced when using PT. However, it was found that long-term compliance was less with PT, and close follow-up of patients for compliance was required, especially in patients with moderate-to-severe OSA [46]. PT adequately related to patient body structure must be used for the success of the treatment. Even when commercial waistbands are not accessible, a custom-made version done by the patients themselves proves to be functional due to individualized size and design. However, everyday disparity and change or advancement of the preliminary results must be considered, and frequent follow-up is optimal [48].

Smart devices for lifestyle enhancement

With the advent of technology, new smart devices are increasingly used by people on a day-to-day basis. These devices, such as smartphones with external sensors and smartwatches with inbuilt sensors, facilitate the detection of the patients' sleep behavior. Traditional visual scoring or sleep-analyzing software is required to assess the smart devices' signal data. Wireless data communication enables uploading data to a cloud database and connected to the individual-health repository [49]. Recently, studies have been aimed at questioning the efficacy of fitness-tracking systems. Schlomann et al. investigated the relationship between physical activity tracking and subjective satisfaction of fitness. According to this study, it has been indicated that tracking systems have the ability to amplify fitness strategies in older adults. However, the tracking function negatively influenced people who were not physically active [50].

Summary

Obesity is extensively linked to the risk of developing OSA, and weight loss has marked improvement in life quality; successful diet incorporation through comprehensive education and exercise programs helps patients achieve positive AHI and BMI changes. Despite the uncertain association between smoking and sleep apnea, smoking cessation can improve OSA symptoms and prevent metabolic disease progression; heavy drinking has been associated with the severity of OSA, and several studies are focused on the synergism between alcohol intake and OSA. PT using at-home tennis ball frameworks considerably reduces severity for supine-mediated OSA. smart devices have become unique gadgets for tracking lifestyle interventions and are continually innovating.

## Conclusions

Treatment for OSA depends on addressing the underlying cause of the disease. As several lifestyle changes have been applied to the successful control of weight loss and metabolic diseases, a comprehensive behavioral intervention addressing OSA therapy, including smoking cessation and alcohol intake, may improve the quality of life for the patients suffering from OSA. Prevention is pivotal to prevent harm and reclaim the quality of life when it comes to sleep and weight. With an appropriate treatment plan, sleep apnea carries a remarkable prognosis. Therefore, it is crucial to see a physician for an accurate diagnosis and begin to control weight.
